# Comparison of a standard CO_2_ pressure pneumoperitoneum insufflator versus AirSeal™: study protocol of a randomized controlled trial

**DOI:** 10.1186/1745-6215-15-239

**Published:** 2014-06-20

**Authors:** Ruzica Rosalia Luketina, Michael Knauer, Gernot Köhler, Oliver Owen Koch, Klaus Strasser, Margot Egger, Klaus Emmanuel

**Affiliations:** 1 Department of General and Visceral Surgery, Krankenhaus der Barmherzigen Schwestern Linz, Seilerstätte 4, Linz 4020, Austria; 2 Department of Anaesthesiology, Krankenhaus der Barmherzigen Schwestern Linz, Seilerstätte 4, Linz 4020, Austria; 3 Department of Laboratory Medicine, Krankenhaus der Barmherzigen Schwestern Linz, Seilerstätte 4, Linz 4020, Austria

**Keywords:** Pneumoperitoneum, AirSeal™, Laparoscopy, CO_2_ Insufflator, Shoulder pain

## Abstract

**Background:**

AirSeal™ is a novel class of valve-free insufflation system that enables a stable pneumoperitoneum with continuous smoke evacuation and carbon dioxide (CO_2_) recirculation during laparoscopic surgery. Comparison data to standard CO_2_ pressure pneumoperitoneum insufflators is scarce. The aim of this study is to evaluate the potential advantages of AirSeal™ compared to a standard CO_2_ insufflator.

**Methods/Design:**

This is a single center randomized controlled trial comparing elective laparoscopic cholecystectomy, colorectal surgery and hernia repair with AirSeal™ (group A) versus a standard CO_2_ pressure insufflator (group S). Patients are randomized using a web-based central randomization and registration system. Primary outcome measures will be operative time and level of postoperative shoulder pain by using the visual analog score (VAS). Secondary outcomes include the evaluation of immunological values through blood tests, anesthesiological parameters, surgical side effects and length of hospital stay. Taking into account an expected dropout rate of 5%, the total number of patients is 182 (n = 91 per group). All tests will be two-sided with a confidence level of 95% (*P* <0.05).

**Discussion:**

The duration of an operation is an important factor in reducing the patient’s exposure to CO_2_ pneumoperitoneum and its adverse consequences. This trial will help to evaluate if the announced advantages of AirSeal™, such as clear sight of the operative site and an exceptionally stable working environment, will facilitate the course of selected procedures and influence operation time and patients clinical outcome.

**Trial registration:**

ClinicalTrials.gov NCT01740011, registered 23 November 2012.

## Background

Laparoscopic surgery is currently actively used in a variety of surgical conditions and is performed through the creation of a workspace between the abdominal wall and the internal organs, most commonly by the insufflation of carbon dioxide (CO_2_) to the level of positive intra-abdominal pressure tolerated by the patient. CO_2_ is the most frequent employed medium because it is relatively inexpensive, colorless, odorless, nonflammable and rapidly eliminated from systemic circulation [1,2]. A good exposure to the operative field facilitates an improved technical performance and is a factor that affects the duration of an operation and the safety of a patient during the course of the procedure. Conventional CO_2_ insufflation systems often respond with a delay to increased intraoperative pressure loss. The collapse of the abdominal cavity during increased systemic absorption of CO_2_ gas, for example as a result of suction or smoke evacuation, may prolong operation time and can be prevented only by an increase in CO_2_ insufflation pressure. The CO_2_ and higher abdominal pressure adversely affects the patient’s homeostasis, causing significant changes in cardiovascular and respiratory systems, decreasing perfusion in abdominal organs and blood flow in the inferior vena cava and causing an increased risk of thrombotic disease [3].

Insufflation of CO_2_ during laparoscopic surgery leads to postoperative shoulder pain. The origin of shoulder pain is commonly assumed to be due to the overstretching of the diaphragmatic muscle fibres owing to a high CO_2_ pressure [4]. The physiology of the pneumoperitoneum is complex with local and systemic effects of a gas instilled under pressure [5]. It has been proven that surgical trauma is connected with the changes in cytokine concentration. Excessive production of pro-inflammatory cytokines after surgical procedures may have an unfavorable influence on the healing process and cause complication during postoperative period. Many physiological changes of laparoscopic surgery have recently been reported in research articles [6-8]. Among these studies, it has been theorized that conventional laparoscopic surgery under CO_2_ gas is immunologically superior to laparotomy [9]. Substantial experimental and clinical evidence also exists indicating that the immune response is better preserved after laparoscopic surgery [9-14], but the mechanism for immune system preservation under the CO_2_ gas-specific effect is still unclear in all situations. To examine changes in the concentrations of certain cytokines and angiogenic factors serum levels in the early postoperative period after laparoscopic surgery further studies are needed. Additionally, the absorption of CO_2_ during pneumoperitoneum can lead to an increase in PaCO_2_ level, however hypercapnia is commonly avoided by appropriate ventilatory changes. Absorption of CO_2_ also alters the acid–base balance and the increase in CO_2_ excretion load. Compared with baseline values, the increased intra-abdominal pressure during pneumoperitoneum can reduce femoral venous flow, intraoperative urine output, portal venous flow, respiratory compliance and cardiac output [3].

Recently, with AirSeal™, a novel class of valve and membrane-free insufflation and/or trocar system is available that responds immediately to the slightest changes in intra-abdominal pressure, maintaining a stable pneumoperitoneum and continuous smoke evacuation even under difficult surgical conditions and constant suction, ensuring visibility. This insufflation system is associated with reduced CO_2_ use, absorption and elimination. Although the reduction of CO_2_ absorption makes this new insufflation system an attractive alternative to standard insufflation systems, to the best of our knowledge, no randomized clinical studies have been performed which characterize the potential benefits and advantages in order to demonstrate significant clinical findings until now.

## Methods/Design

### Objectives and hypotheses

The primary objective of the present randomized trial is to investigate the mean operative time and to study the frequency and intensity of postoperative shoulder pain in patients undergoing laparoscopic cholecystectomy, colorectal surgery, and hernia repair with AirSeal™ (group A) compared with a standard pressure CO_2_ insufflator (group S).

The first primary endpoint will be whether the time of surgery in group A is more than or equal to the time of surgery in group S (H0_PE1_) or whether the time of surgery in group A is less than the time of surgery in group S (H1_PE1_).

The second primary endpoint will be whether shoulder pain in group A is more than or equal to shoulder pain in group S (H0_PE2_) or whether shoulder pain in group A is less than shoulder pain in group S (H1_PE2_).

### Study population and location

The study population will consist of patients undergoing elective laparoscopic cholecystectomy, colorectal surgery and hernia repair. A clinical evaluation and pre-randomization assessment must be completed for every patient, including a review of the eligibility criteria, signed and dated informed consent, inquiry of relevant past medical history and anesthesiological preoperative assessment including American Society of Anesthesiologists (ASA) class. Dropout criteria will be if patients recruited for laparoscopic resection are converted to an open procedure. Detailed eligibility criteria are listed in Table 1.

**Table 1:** Eligibility criteria

**Inclusion criteria**	**Exclusion criteria**
• Aged 18 years and over	• Previous extensive abdominal surgery
• ASA ≤ 3	• Acute surgical intervention
• Informed consent provided	• Immunological dysfunctions like advanced liver disease, HIV or hepatitis C virus infection
• Diseases of the gallbladder, colon or hernias treated with elective laparoscopic procedure	• Pregnancy and lactation

Patients who meet the eligibility criteria will be given a patient information sheet and time to consider the trial. If interested, informed consent will be obtained for each patient prior to randomization. All personal information obtained for the study will be held securely and treated as strictly confidential.

This single centre trial will be performed at a high volume institution with experience in laparoscopic surgery (Department of General and Visceral Surgery, Sisters of Charity Hospital Linz, Austria). The clinical study centre of the surgical department will conduct this trial.

### Study design and randomization

After screening for eligibility is performed and informed consent is obtained, patients will be randomly stratified regarding type of operation in a 1:1 ratio into one of the following study arms: laparoscopic surgery with an AirSeal™ CO_2_ pressure insufflator (Surgiquest Inc., Milford, USA) (group A, experimental arm) or laparoscopic surgery with a standard CO_2_ pressure insufflator (Olympus America Inc. UHI-3, Center Valley, PA, US) (group S, control arm).

Patients are randomized using a web-based, central randomization and registration system. Treatment according to randomization (operation with AirSeal™ or standard CO_2_ pressure pneumoperitoneum) must be carried out within 14 days after randomization. For the study flow of the trial according to CONSORT guidelines, please see Additional file 1.

### Primary endpoints

Operation time, defined as the time from skin incision to closure of wound in minutes, is routinely documented in our operation program and will be evaluated after the operation by a newly designed survey. Postoperative shoulder pain will be assessed within 48 hours after the operation using the visual analogue pain scale (VAS). Postoperative pain will be assessed in a double-blinded manner, neither the patient, the assessor of shoulder pain, nor the postoperative caregivers will be aware of the technique to which the patient will been randomized.

### Secondary endpoints

Blood sample remains will be collected during routinely preformed venepuncture prior to surgery, intraoperatively after pneumoperitoneum skin incision, after skin suture and 24 hours postoperatively. They will be kept and used for immunological evaluation. All routine laboratory diagnostics will be performed immediately after blood collection. For the determination of the target parameters the EDTA (Ethylenediaminetetraacetic acid) plasma must be frozen by -80°C. All interleukins and cytokines will be measured at the end of patient recruitment in one sequence. Blood gas analysis and documentation of the ventilation volume will be performed. This intraoperative data will be also collected by the survey. Analgetic requirements, wound infections, anastomotic leakage and postoperative hemorrhage of all the patients in the postoperative period and length of hospital stay will be also recorded.

The immunological aspects will be: changes in the concentration of certain cytokines and angiogenic factors serum levels in the early postoperative period, and target parameters (soluble interleukin-1 receptor family member ST2 (sST2), interleukin (IL)-6, IL-8, IL-10, vascular endothelial growth factor A (VEGF-A) and endostatin).

The anesthesiological aspects will be: tidal volume, positive end expiratory pressure (PEEP), peak pressure, respiratory minute volume, acid–base balance and CO_2_ elimination in the patients, measured before insufflation, during surgery and after deflation of the pneumoperitoneum.

### Operative procedure

All patients will be anesthetized following a strict protocol for premedication, anaesthesia and intubation as well as mechanical ventilation and monitoring for CO_2_ level. The operation will be performed by experienced staff surgeons involved in the study. In all patients, access will be achieved using four working ports and the same instruments. In group A, one 5 mm AirSeal™ access port will be used instead of one standard 5 mm port. Pneumoperitoneum will be created without a visual control using a Veress needle (Ethicon Endo Surgery Inc., Cincinnati, US) for insertion through a small skin incision in the umbilical region. Intraperitoneal pressure using CO_2_ gas should be kept at around 12 mmHg. The patient should be placed in a reverse Trendelenburg position with left side down. Residual CO_2_ pneumoperitoneum will be evacuated at the end of the procedure by compressing the abdomen whilst taking care to keep the valve of the trocar open.

### Data collection

Daily visits with study patients will be done by the clinical investigator to collect information on primary and secondary outcome parameters. A detailed database will be collected, including sex, age and body mass index (BMI), ASA grade, medical history prior to operation, quality of surgical field exposure, duration of surgery and postoperative shoulder and other pain VAS score. The number of intravenous and oral medications required, use of drainage and other intraoperative and postoperative events in all patients will be recorded as well as length of hospital stay. All routine blood parameters and blood gas analysis data will be saved. Table 2 summarizes the course of examination and data collection process.

**Table 2:** Course of examination

**Visit**	**Screening visit**	**At skin incision (pneumoperitoneum insufflation)**	**At skin suture (pneumoperitoneum deflation)**	**Daily postoperative ward visits**	**At discharge**
Past and current medical history	x				
Preanesthesia evaluation	x				
Physical examination and personal data (height, weight, age, ASA)	x				
Randomization	x				
Blood draw and frozen sample	x	x	x	x	
Blood gas draw		x	x		
VAS shoulder pain				x	
Postoperative complications (wound infections, intra-abdominal abscess and anastomotic leakage)				x	x

### Ethical considerations

Prior to the start of the trial, protocol written approval was authorized by the local ethical committee of the Hospital of the Sisters of Charity in Linz, a member of the independent Ethics Committee (IEC) of Austria (Study number: 28/12 AirSeal™ Trial).

Following IEC approval, all subsequent protocol amendments and changes to the informed consent document must be approved by the responsible IEC. The IEC must be informed of the end of the trial. The investigator must keep a record of all communications with the IEC and the regulatory authorities.

### Patients’ confidentiality

It is the responsibility of the investigator to maintain patient confidentiality. During the trial patients will be identified solely by means of their year of birth and individual identification code (screening number plus randomization number). Trial findings will be stored in accordance with local data protection law and ICH GCP (International Conference on Harmonisation of Technical Requirements for Registration of Pharmaceuticals for Human Use provide a unified standard for the European Union, Japan, and the United States to facilitate the mutual acceptance of clinical data by the regulatory authorities in these jurisdictions) guidelines and will be handled in strictest confidence. The investigator will maintain a personal subject identification list (screening numbers with the corresponding subject name) to enable records to be identified.

### Patient protection

The responsible investigator will ensure that this study is conducted in agreement with the Declaration of Helsinki (Tokyo, Venice, Hong Kong, Somerset West and Edinburgh amendments).

## Informed consent

All patients recruited for the study will be informed of the aims of the study, collection of blood sample remains taken during routine pre- and postoperative venepuncture for immunological evaluation, the procedures and possible hazards to which he/she will be exposed and the mechanism of treatment allocation. Patients are free to withdraw from the study at any time without providing any specific reason. Furthermore, it is the responsibility of the investigator to explain to patients their duties within the trial. They will be informed about the strict confidentiality of their patient data but that their medical records may be reviewed for trial purposes by authorized individuals other than their treating physician.

### Trial insurance

This trial is comparing two standard treatments that represent current medical practice in Austria. No experimental treatment is delivered to patients within this trial. The trial is not subject to the Austrian Drug Law and may be considered as a so-called ‘free’ clinical study, for which no legal requirements exist concerning patient indemnity insurance. This trial is regulated by the Austrian Medical Device Law § 40.5. The sponsor has procured public liability insurance from the Sisters of Charity Hospital Linz that covers the conduct of clinical trials.

### Statistical considerations

From a statistical point of view the present trial shows a confirmatory status, superiority approach and a parallel group design (group A with AirSeal™ use versus group S with standard CO_2_ pressure pneumoperitoneum insufflator use). The type I error preservation by the gatekeeping approach (primary endpoint one: time of surgery (in minutes) and primary endpoint two: shoulder pain at 48 hours after end of surgery (measured using VAS)) was calculated. Stratification according to the type of surgery was done.

### Sample size estimation

On the basis of a comparison of two proportions, a sample size of 182 (n = 91 per group) is needed to have 80% power at a 5% significance level, assuming a dropout rate of 5%.

Data, assumptions and conditions are: type I error (2.5% one-sided), type II error (20%), non-parametric test (Mann–Whitney *U*-test), n (group A): n (group S) (ratio of 1:1) and per-protocol analysis:

(1) $$\begin{array}{l} \mathrm{group}\;A\;\left(\mathrm{mean}\;\pm \;\mathrm{standard}\;\mathrm{deviation}\right):\;74,8\;\pm \;41,3\;\min\\ \mathrm{group}\;S\;\left(\mathrm{mean}\;\pm \;\mathrm{standard}\;\mathrm{deviation}\right):\;93,5\;\pm \;41,3\;\min \end{array}\;\}\Delta =20\%$$

### Analysis populations

The intention-to-treat (ITT) population: all subjects in whom the use of AirSeal™ or a standard CO_2_ pressure pneumoperitoneum insufflator has already begun will be included in the ITT population. All variables will be analyzed. The ITT population will be used for the primary safety evaluation of implantation (ITT analysis).

The per-protocol (PP) population will be: all subjects without occurrence of dropout situation (all valid cases) will be included in the PP population. All variables will be analyzed. The PP population will be used for the primary efficacy evaluation (PP analysis).

### Handling of implausible values and missing values

Implausible values have to be identified by the clinical investigator. They will be converted into missing values. Missing values will not be replaced, with the following exception: missing values at the two primary endpoints will be replaced by the mean of all plausible data (both groups) of the respective endpoint. The missing-value replacement procedure described above will be only applied to the ITT analyses (no replacement of missing values in the PP analysis).

### Statistical methods

The estimation of effect size for selected variables will be the calculation of two-sided 95% confidence intervals.

Primary endpoint one will be set due to the clear duration difference between colorectal surgery and the other types of surgery (laparoscopic cholecystectomy and laparoscopic hernia repair) a normal distribution of the data can be excluded. Therefore, a nonparametric test, namely the exact Mann–Whitney *U*-test will be used (type I error = 2.5% one-sided).

Primary endpoint two (only if H0_PE1_ is rejected), will be set by checking data sets for normal distribution (test of normality: Kolmogorov-Smirnov with Lilliefors significance correction, type I error = 5%). In the case of normal distribution the *t*-test for independent samples with correction for heteroscedasticity (Levene’s test, type I error = 5%) will be applied. In the case of a significant deviation from the normal distribution the exact Mann–Whitney *U* test will be used instead. In both cases the type I error will be 2.5% one sided.

Metric variables data sets will be checked for normal distribution (test of normality: Kolmogorov-Smirnov with Lilliefors significance correction, type I error = 5%). Normally distributed data sets will be compared between the groups by the *t*-test for independent samples with correction for heteroscedasticity (Levene’s test, type I error = 5%). If there is a significant deviation from the normal distribution the exact Mann–Whitney *U* test will be used instead.

Ordinal variables will be checked by the exact Mann–Whitney *U* test and nominal variables by the exact chi-square homogeneity test or, for 2 × 2 frequency tables the Fisher's exact test.

All tests will be two-sided with a confidence level of 95% (*P* <0.05). As no adjustment of the *P* values will be made, the resulting *P* values will only be descriptive.

### Presentation of the results (descriptive analysis and graphs)

Nominal variables will be presented using counts and percentages. Ordinal variables will be presented using counts and percentages (where appropriate) or minimum, 25% percentile, median, 75% percentile and maximum and number of patients (where appropriate). Metric variables will be presented using minimum, 25% percentile, arithmetic mean, median, 75% percentile, maximum, standard deviation and number of patients. All results will be presented in the form of tables, selected results additionally in the form of graphs (bar charts and boxplots). Subgroup analyses can be performed for cause, however, all statistical results will be only descriptive. No interim analysis is intended.

## Discussion

Many physiological changes occur during CO_2_ pneumoperitoneum. The severity of these pathophysiological disturbances depends on the intra-abdominal pressure being used and patients´ exposure to CO_2_ pneumoperitoneum, hence on operation time. Post-laparoscopic shoulder pain one to three days after laparoscopy is well described [15]. There have been few reports comparing low and high pressure pneumoperitoneum, demonstrating that low pressure was associated with lower postoperative incidence of shoulder pain [16,17], while others have shown that pressure had less effect on pain [18]. AirSeal™, a novel class of valve-free insufflation system, works by a pressured gas barrier of forced insufflation rather than the trapdoor valves used by standard insufflators [19] which, in a non-randomized clinical study in patients undergoing laparoscopic renal surgery, was reported to be associated with less carbon dioxide use, decreased blood loss and shorter operation time than traditional insufflation [20]. The maintenance of pneumoperitoneum during changes in equilibrium may be a factor during critical moments, such as active bleeding. Within this trial we will evaluate the potential clinical advantages and benefits of AirSeal™ in patients selected for elective laparoscopic cholecystectomy, colorectal surgery or hernia repair.

## Trial status

This trial is currently recruiting. The last patient is expected to be fully recruited by March 2014.

## Abbreviations

ASA: American Society of Anesthesiologists; BMI: Body mass index; CO_2_: Carbon dioxide; HIV: Human immunodeficiency virus; IEC: Independent Ethics Committee; IL: Interleukin; PaCO_2_: Arterial carbon dioxide partial pressure; PEEP: Positive end expiratory pressure; sST2: Soluble interleukin-1 receptor family member ST2; VAS: Visual analog score; VEGF-A: Vascular endothelial growth factor A.

## Competing interests

The authors declare that they have no competing interests.

## Authors’ contributions

RL conceived the study and wrote the study protocol and manuscript. MK helped to develop the statistical design. KE and MK provided scientific input and reviewed the manuscript. OK and GK undertook acquisition of data. KS wrote the anesthesiological, ME designed the laboratory medicine protocol for the trial. All authors read and approved the final manuscript.

## Supplementary Material

Additional file 1 Study flow of the trial according to CONSORT guidelines. Click here for file
