# Supplementary material for: Comparison of a standard CO2 pressure pneumoperitoneum insufflator versus AirSeal™: study protocol of a randomized controlled trial
Source: Trials. 2014 Jun 20;15:239. doi: 10.1186/1745-6215-15-239 (PMC4078359; doi:10.1186/1745-6215-15-239)
Supplement: Additional file 1 — Study flow of the trial according to CONSORT guidelines. [file 1745-6215-15-239-S1.docx]

Additional file 1.

## Follow-Up

Analysed

(n=x)

## Analysis

Analysed

(n=x)

Discontinued intervention (conversion to open surgical procedure)

(n=x)

Discontinued intervention (conversion to open surgical procedure)

(n=x)

## Enrollment

Laparoscopic surgery with AirSeal^®^

(n= 91)

## Allocation

Laparoscopic surgery with standard insufflator (n= 91)

Excluded (n=x)

♦  Not meeting inclusion criteria (n=x)

♦  Declined to participate (n=x)

Assessed for eligibility (n=x)

**CONSORT Study flow for AIRSEAL^®^ Trial**

Randomized (n= approx. 182)
